# An evaluation of physical activity levels and mental health among young people: a cross-sectional study

**DOI:** 10.1186/s40359-025-02533-2

**Published:** 2025-03-05

**Authors:** Gökhan Çakir, Utku Isik, İsa Kavalci

**Affiliations:** 1 Faculty of Sport Sciences, Recep Tayyip Erdogan University, Rize, Türkiye; 2 Faculty of Sport Sciences, Ege University, Izmir, Türkiye

**Keywords:** Physical activity, Psychological resilience, Psychological vulnerability, Mental well-being

## Abstract

**Background:**

The aim of this study was to determine the role of physical activity on mental health. This topic is essential, as physical activity is widely recognized for its potential impact on mental health outcomes, including well-being and resilience. However, there remains a need for further research on how specific types and levels of activity contribute to mental health, particularly among young people.

**Methods:**

The study group consisted of 427 students studying at universities in Turkey. Data were collected through the Personal Information Form, the International Physical Activity Questionnaire-Short Form, the Warwick-Edinburgh Mental Well-Being Scale Short Form, the Psychological Resilience Scale (Short Form), and the Psychological Vulnerability Scale. Skewness and kurtosis values were used to analyze the normality of data distribution. MANOVA, Chi-Square Test, Pearson correlation coefficient, and Multiple Stepwise Regression tests were conducted for data analysis.

**Results:**

The results showed that female students were more inactive and engaged in less vigorous activities than males (*p* < 0.05). In contrast, the proportional distribution of physical activity levels by grade level was similar (*p* > 0.05). Descriptive findings indicated that students displayed relatively high levels of psychological vulnerability. Mental health components were found to be interrelated (*p* < 0.05). Physically active students exhibited higher levels of mental well-being and psychological resilience and lower levels of psychological vulnerability (*p* < 0.05). Finally, walking emerged as the best predictor of students’ psychological resilience and mental well-being, and along with moderate levels of physical activity, it significantly contributed to improved mental well-being (*p* < 0.05).

**Conclusions:**

This research shows that participation in physical activity increases university students’ mental resilience, reduces their psychological vulnerability and supports their mental well-being. In particular, walking and moderate physical activity were found to have the strongest effects. The results emphasise that physical activity promotion is a critical requirement for improving students’ mental health.

## Background

Many students see university life as a challenging period. While students pursue positive goals such as building strong relationships, meeting people from different cultures, and achieving academic success [1], they also encounter stressors such as depression and anxiety associated with personal growth and adaptation to new environments [2]. Nevertheless, when students start a new experience at the beginning of their university life, their habits such as PA, alcohol and tobacco use may change and this may affect their health behaviours in the future [3, 4]. Studies indicate that university students may face greater psychological challenges compared to their peers outside higher education, showing more depressive symptoms and increased stress due to factors like being away from home, forming new friendships, and academic demands [5–7]. Therefore, it is essential for university programs to take an active role in developing preventive and supportive interventions to protect students’ mental health [8, 9].

## Introduction

Students’ psychological resilience, mental well-being and psychological vulnerability can be considered as important concepts in stressful situations encountered during university years [10]. Moreover, these concepts are among the important indicators of a person’s mental health [11]. While psychological resilience and mental well-being are characteristics associated with maintaining a positive emotional state, signs of psychological vulnerability, such as heightened sensitivity to stress or difficulty in coping with challenges, may indicate that an individual is experiencing negative emotions [12]. Furthermore, studying both positive mental health and mental illness is necessary to fully comprehend mental health [12]. According to the World Health Organization (WHO), mental health is “a state of well-being in which the individual realizes his or her own abilities, can cope with the normal stresses of life, can work productively and fruitfully, and is able to make a contribution to his or her community” [13]. While depression, anxiety, and psychological distress are markers of mental disease, positive affect, life satisfaction, and psychological well-being are examples of constructs representing positive mental health [12].

Psychological resilience is the strength of the individual which connects him/her more firmly to life, makes it easier for him/her to cope with the difficulties in life, allows him/her to lead a healthier and worry-free life, and makes him/her stronger [14]. A psychologically healthy person is someone who can withstand negative events without easily falling apart and can recover quickly afterward. In this respect, resilience can be seen as an important determinant of quality of life and can be associated with improved, functional, physical and psychological well-being [15]. On the other hand, university students who have to be separated from their friends and social environment feel more lonely, receive less social support, and thus, their psychological resilience may be negatively affected [16, 17].

Mental well-being, another variable in this study, is a term often used interchangeably with “positive mental health” in both policy and academic literature [18]. Research shows that individuals with higher levels of positive mental health have a lower incidence of physical disease, experience less conflict in their relationships, are more productive, and are less likely to suffer from psychiatric disorders like depression and anxiety [19]. In this context, it is important to consider that various challenges and significant life changes—such as transitions into adulthood, unexpected life events, and health crises like epidemics—can negatively impact students’ mental well-being during their university years [19]. In addition, mental well-being can be explained as a concept that generalises the definitions of well-being in the literature. Therefore, it can be said to have a strong link with psychological resilience [19].

Psychological vulnerability is one of the main variables that may affect the mental well-being of individuals [20]. Individuals may experience stress and anxiety in response to vulnerabilities that can disrupt their social, physical, and psychological functioning [21]. Hence, it can be said that people with a low level of psychological vulnerability will face fewer psychological problems [22]. University students can also be considered to have a fragile (weak mental health) structures. This is partly due to various accumulated problems such as university pressure, programme changes, paid work activities, financial difficulties and isolation [23].

The recognition of physical activity (PA) as a crucial factor for enhancing students’ mental health is growing due to its proven benefits and increasing awareness of mental health issues [24]. Today, only 28% of adults aged 18 years and over meet the physical activity qualifications set by the World Health Organisation [24]. Similarly, in Türkiye, 81% of young people are not sufficiently physically active: [25]. These statistical data reveal the striking facts about the PA levels of students. WHO emphasises the importance of being physically active in the Global Action Plan for Physical Mobility covering the years 2018–2030 [13]. Likewise, considering the positive effects of physical activity on psychological health stated in numerous studies, the importance of being physically active becomes even more evident.

This study suggest that the mental health of university students who are sufficiently physically active may be at better levels compared to those who are minimally physically active and/or physically inactive. In this context, this study seeks to examine how the mental health of students varies with their levels of PA and whether there is a significant relationship between PA and mental health outcomes. The other question that this study seeks to answer is which type of PA (walking, moderate, vigorous) will be a significant predictor of mental health.

The main objectives of this study are as follows:

1. To examine the relationships between psychological resilience, mental well-being and psychological vulnerability levels of university students and to analyse which variables are related to the PA levels.
2. To determine whether the mental health (psychological resilience, mental well-being and psychological vulnerability) of students with varying PA levels differ.
3. To analyse which type of PA is a better predictor of mental health.

### Literature review

Studies in the literature show that people with high levels of psychological vulnerability have significantly lower life satisfaction, reduced psychological resilience, poorer psychological coping resources and more frequent negative emotions [26, 27]. A meta-analysis revealed that psychological resilience had a positive correlation with positive health indicators and a negative correlation with bad health indicators. Additionally, it is anticipated that people with strong psychological resilience will also have high levels of mental well-being, whereas people with low psychological resilience will probably have low levels of mental well-being [28].

In this sense, it is predicted that there may be a relation between mental well-being, psychological resilience and psychological resilience of university students.

Physical activity improves psychological health and well-being. Physical activity also improves quality of life [29]. In a sample of university students, Škrlec et al. looked at the association between negative emotions (stress, anxiety, and sadness) and physical activity [30]. They came to the conclusion that physical exercise may lessen bad feelings. Casimiro-Andújar et al. stated that participating in a physical activity programme has a positive effect on the working environment and can improve the general health and mood of individuals [31]. Similarly, Granero-Jiménez et al. reported that higher levels of physical activity were associated with higher levels of psychological health in their study on a sample of students aged 18–35 years [32]. In his study on university students, Herbert reported that physical activity was positively associated with mental health and well-being [33]. The results also showed that workouts that include several weeks of low to moderate intensity aerobic exercises can improve students’ mental health.

According to all of this research, it is expected that university students’ mental health may alter depending on their degree of physical exercise. It is also hypothesized that the optimal indicator of mental health may vary based on physical activity level.

Regular physical activity plays a key role in health and well-being [34]. Recognising the relationship between physical activity, psychological resilience and the outcome of depressive symptoms would be an important step in developing a healthy lifestyle for young people [35]. Dunston et al. in their study conducted on university students examining the relationship between physical activity intensity and psychological resilience, reported that intense physical activity and psychological resilience scores were positively correlated [36]. Similarly, in different research looking at the connection between university students’ levels of physical exercise and psychological resilience, physical activity level was expressed as a variable predicting the level of psychological resilience [37].

It is anticipated that university students’ psychological resilience levels may alter based on their levels of physical exercise, in accordance with all of this research. It is also assumed that the best predictor of psychological resilience may differ depending on the intensity of physical activity.

Charbonnier et al. in their longitudinal study examining the psychological vulnerability of university students, stated that the level of vulnerability in students was found to be significantly high and that these findings were worrying for the future [38]. In their study on the connection between stress vulnerability and physical activity in college students, Xu et al. found a substantial inverse link between depressive symptoms and physical activity [39]. The importance of physical activity is better understood considering the fact that individuals with high psychological vulnerability are not resistant to traumatic events. As a matter of fact, a scientific study has shown that children with high physical activity levels can cope better with traumatic events compared to children with low physical activity levels [40].

In line with the limited data obtained, it is assumed that the psychological vulnerability levels of university students may vary according to different physical activity levels.

### Hypotheses

Based on the literature review, the following hypotheses are proposed:

#### H_1_

There are statistically significant relations between students mental well-being, psychological resilience, and psychological vulnerability.

#### H_2_

Mental well-being levels of students with varying physical activity levels differ.

#### H_3_

Engaging in walking and moderate intensity physical activity significantly predicts mental well-being.

#### H_4_

Students’ psychological resilience levels differ based on their levels of physical activity.

#### H_5_

Engaging in vigorous physical activity is a significant predictor of psychological resilience.

#### H_6_

Students’ psychological vulnerability levels differ based on their levels of physical activity.

## Method

### Research model

The concept of “mental health” was considered as a composite structure covering psychological resilience, psychological vulnerability and mental well-being. In accordance with the structure of the International Physical Activity Questionnaire, PA levels were categorised as inactive, minimally active, and HEPA (health enhancing physical activity; a high active category) [41]. In addition, PA types were categorised as walking, moderate intensity and vigorous intensity as continuous measurements.

**Fig. 1:**
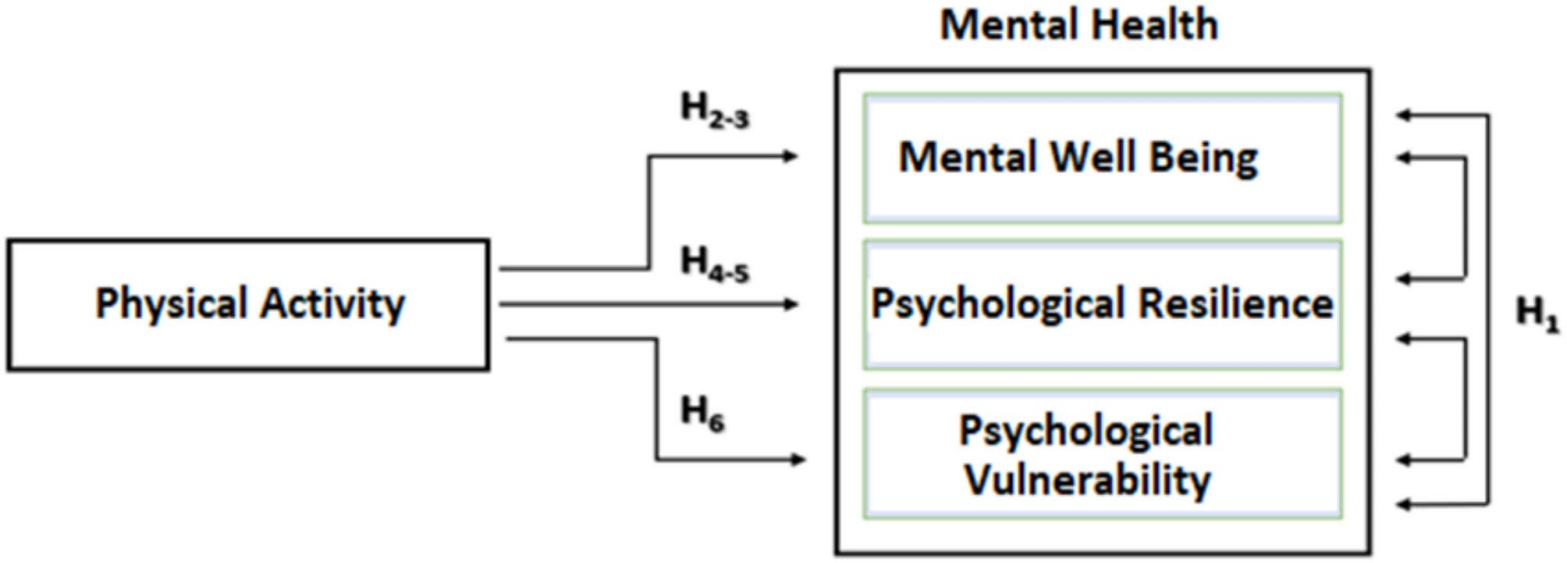
Research model

The current study was created using a quantitative research framework using a descriptive-relational survey methodology. The conceptual model developed to determine the role of physical activity on mental health (mental well-being, psychological resilience and psychological vulnerability) is shown in Fig. 1.

### Procedure, ethics and recruitment

The data were obtained from undergraduate students enrolled in Recep Tayyip Erdogan University (RTEU) in the spring semester of 2023–2024, collected between February 5 and February 12, 2024. All data were collected within this time period. In addition to the demographic information of the students (gender, age, faculty and grade level), physical activity, psychological resilience, psychological vulnerability and mental well-being scales were transferred to the web-based Google forms as well. The links of the surveys were distributed to the students via social media. It was ensured that participation was voluntary. We acquired informed consent and permission from each individual. The goal of the study was briefly explained to the participants. Questionnaires with missing data were not accepted. The study was approved by the Recep Tayyip Erdogan University Social and Human Sciences Ethics Committee (number: 2023/132, Date: 30/03/2023).

### Population-sample

A total of 462 students participated in the study. The study did not include the responses of 15 pupils (3.2%) whose questionnaires were discovered to have been filled out inaccurately or inconsistently. The study’s statistical tests are sensitive to extreme values; thus, 20 replies (4.4%) were removed from the data after checking the descriptive table’s 5% trimmed values. Responses of 427 students were finally included in the study.

In this cross-sectional study, convenience sampling was used. To calculate the necessary sample size, the G*Power (3.1.9.7, Germany) software [30] was utilized. A sample size of 210 individuals is adequate for research with a real power of 0.95, according to G*power. In addition, the population-sample table was used to determine the sample size. The population of the study consists of 16,965 students enrolled in Recep Tayyip Erdoğan University (RTEU) in the spring semester of 2023–2024. According to Cohen et al. a minimum of 377 participants are required for sample representation at a 95% confidence level and a 5% confidence interval if the population has 20,000 members [42]. In this context, it is thought that the representative power of the sample size (*n* = 427) will be sufficient.

Inclusion Criteria Of Participants: Voluntary participation in the study, being a university student, being a male or female student, to be registered at Recep Tayyip Erdoğan University. Exclusion Criteria Of Participants: Not being a university student, not participating in the study voluntarily.

### Data collection tools

#### International physical activity questionnaire-short form (IPAQ-SF)

The validity and reliability study of the questionnaire form was carried out by Craig et al. [43] and its Turkish adaptation was carried out by Öztürk [44]. There are seven questions in all, each of the seven questions is designed to evaluate physical activity levels during the previous week. The form asks about how much time people spend engaging in vigorous and moderate physical activity, as well as passive activities like sitting and strolling. By computing metabolic equivalents (METs), this data is acquired. 1.5 MET for sitting, 3.3 MET for walking, 4 MET for moderate physical activity, and 8 MET for vigorous physical activity were found to be the respective MET values. As a result, these numbers allow for the calculation and evaluation of each person’s weekly physical activity level through repeated assessments. After determining the MET value, it is possible to classify the physical activity levels of individuals as inactive (< 600 MET), minimally active (600 MET-3000 MET) and HEPA active (> 3000 MET) [44]. Furthermore, the Turkish version of IPAQ-SF has been demonstrated to be valid and reliable in determining physical activity levels. Structural, concurrent, and criterion validity, as well as test-retest reliability, were evaluated in Öztürk’s study. Test-retest reliability coefficients were found to be *r* = 0.69 for the short form and *r* = 0.64 for the long form, while criterion validity coefficients were *r* = 0.30 for the short form and *r* = 0.29 for the long form. These findings confirm that the Turkish version of IPAQ-SF provides consistent and comparable data for assessing physical activity [44].

#### Warwick-Edinburgh mental wellbeing scal- short form (WEMWB-SF)

The validity and reliability tests of the scale were conducted by Stewart-Brown et al. [45], and its adaptation into Turkish was carried out by Demirtaş and Baytemir [46]. The scale is a 5-point Likert type scale and consists of 7 items. All items in the scale consist of positive statements. The participants were asked to answer according to their recent activities in the last two weeks. Since the validity and reliability of the scale was carried out on Turkish university students, it is considered to be a valid and reliable measurement tool that can be used to measure the mental well-being levels of university students. The Cronbach alpha reliability coefficient of the scale was calculated as 0.86 [46]. In this study, it was determined as 0.80 (Table 1).

**Table 1:** Descriptive statistics of the participants

Variables	Descriptive statistics and reliabilities							Correlations		
	Mean	SD	Skewness	Kurtosis	α	ω		1	2	3
1. PR	2.98	0.77	-0.77	0.31	0.85	0.85		-		
2. MWB	3.46	0.65	0.17	-0.04	0.80	0.81		0.58**	-	
3. PV	2.86	0.75	0.15	-0.33	0.68	0.67		-0.43**	-0.50**	**-**

**P < 0.01; PR = Psychological Resilience, MWB = Mental Wellbeing, PV = Psychological Vulnerability

#### Psychological resilience scale (short form)

Doğan [47], adapted the scale developed by Smith et al. [48] to measure psychological resilience into Turkish. The 5-point Likert type scale consists of 6 items. Items 2-4-6 are coded in reverse order. Since the validity and reliability of the scale was carried out on Turkish university students, it can be said that the scale is a valid and reliable measurement tool that can be used to measure psychological resilience of university students. The internal consistency coefficient of the scale was found to be 0.83 [47] while it was found to be 0.85 in this study (Table 1).

#### Psychological vulnerability scale

Developed by Sinclair and Wallston [49], the scale was adapted into Turkish by Akın and Eker [50]. Consisting of a total of 6 items, the scale does not include any negative items (1 = not at all suitable, 5 = completely suitable). High scores obtained from the scale indicate a high level of psychological vulnerability. The Cronbach alpha internal consistency reliability coefficient of the scale was found to be 0.75 [50]. It was found to be 0.68 in this study (Table 1).

#### Data analysis

Statistical Package for Social Sciences (SPSS) 27.0 programme was used to analyse the data. Preliminary analyses were performed in order to fulfil the assumptions of hypothesis tests before the analyses. Linearity and multicollinearity problem, homogeneity of variance-covariance matrix, and univariate and multivariate normality values were examined for the assumptions of One-Way MANOVA and Pearson Correlation test [51, 52]. The univariate normality of the dependent variable histogram graphs, skewness and kurtosis values, Levene test, and box m test results were also examined. George and Mallery, -2 and + 2 values were taken as reference intervals for skewness and kurtosis values [53]. As a result of the analyses, it was determined that the data showed normal distribution (Table 1). MANOVA test is especially sensitive to extreme values [52]. In order to detect the extreme values, 5% trimmed values in the descriptive table were checked and 20 cases (4.4%) were excluded from the analysis. Some factors were analysed for Stepwise Regression test assumptions. These factors are; no high correlation between independent variables, the number of observations being 20 times higher than the number of independent variables, linear relationship between dependent and independent variables, presence of multicollinearity problems in the data [54] and presence of autocorrelation in the model [55]. No infraction was identified in the preliminary analyses.

Hair et al. states that Mc Donald’s omega values should be 0.6 and above to ensure the reliability of the data obtained from the scales [56]. In the light of this view, it can be said that the data obtained from the survey are reliable. While evaluating the Pearson Correlation coefficients, 0.90-1.00 was considered as very significant, 0.89 − 0.70 as significant (high), 0.69 − 0.40 as moderate, 0.39 − 0.20 as weak (low), and 0.19-0.00 as no relationship [54].

Descriptive statistics and Chi-Square analysis were applied based on the mean scores of the responses received from 427 participants after these preliminary analyses. Subsequently, One-way MANOVA, Multiple Stepwise Regression and Pearson Correlation test were applied as hypothesis tests. In addition, Tukey HSD and Tamhane tests, which are the 2nd level tests used depending on the homogeneity of variances, were applied for the groups in which significant differences were observed in MANOVA analysis. Finally, the data were tested at *p* < 0.05 and *p* < 0.01 significance levels.

## Results

### General characteristics of participants

A total of 427 students (*n* = 290 female, 67.9%, and *n* = 137 male, 32.1%) from 20 different faculties (age; x̄=22.32, SD = 3.97) participated in the study. Of the students, 27.6% were in the first year (118 students), 19.7% in the second year (84 students), 19.3% in the third year (83 students), and 33.3% in the fourth year (142 students).

### Descriptive statistics

Descriptive statistics (normality distributions, reliability analysis and correlation findings) of university students are shown in Table 1.

The values in Table 1 show the mean scores obtained from the scales. Accordingly, the PR mean score of the participants was found to be (x̄ =2.98 ± 0.77), MWB was found to be (x̄=3.46 ± 0.65), and the psychological vulnerability mean score was found to be (x̄=2.86 ± 0.75). When the correlation coefficients between the variables are analysed, it is concluded that there is a moderate positive correlation between PR and MWB (*r* = 0.58, *p* < 0.01), a moderate negative correlation between PR and PV (*r*=-0.43, *p* < 0.01) and a moderate negative correlation between MWB and PV (*r*=-0.50, *p* < 0.01). Chi-square test results for the distribution of gender and class levels of university students according to physical activity levels are shown in Table 2.

**Table 2:** Chi-Square test results of gender and class level according to participants’ physical activity levels

Variables	Inactive		Minimally active			HEPA active		Statistics
	*n*	%		*n*	%	*n*	%	X^2^; *p*
***Gender***								
Female	120	41.3		127	43.7	43	14.8	X^2^ = 37.340
Male	20	14.5		71	51.8	46	33.5	***p*** ** = 0.001**
***Grade***								
1st Year	44	37.2		48	40.6	26	22.0	
2nd Year	29	34.5		40	47.6	15	17.8	X^2^ = 6.728
3rd Year	19	22.8		42	50.6	22	26.5	*p* = 0.347
4th Year	48	33.8		68	47.8	26	18.3	
Total	140	32.7		198	46.3	89	21.0	

The results of the analysis show that 32.7% of the participants were inactive, 46.3% were minimally active and 20.8% were HEPA active in PA participation. It was observed that 33.5% of male participants were HEPA active while only 14.8% of female participants were HEPA active. On the other hand, 41.3% of the female participants were inactive compared to 14.5% of the male participants. Thus, it was determined that there was a statistically significant relationship between gender and PA (X^2^ (df = 2 *n* = 427) = 37.340, *p* < 0.05). In terms of the grade levels, it was concluded that the physical activity levels had a homogeneous distribution and there was no statistically significant relationship in terms of grade levels (X^2^ (df = 6 *n* = 427) = 6.728, *p* > 0.05).

### Hypotheses tests

#### Findings related to hypothesis 2-4-6

Table 3 shows the results of MANOVA test to determine whether physical activity levels have an effect on composite mental health levels.

**Table 3:** MANOVA results of paticipants’ composite mental health levels according to physical activity categories

Wilks’ Lambda	F	Hypothesis Df	Error Df	Statistics
0,917	6,202	6	844	***p*** ** = 0.001**

MANOVA test results on mental health (psychological resilience, mental well-being and psychological vulnerability) reveal that students’ mental health levels differ significantly depending on their PA levels (WilksL(λ) = 0.917; F_(6.844)_ = 6.202; *p* < 0.05). This result suggests that the scores obtained from the linear component varied depending on the PA levels. As the MANOVA test indicated a significant difference, ANOVA test was conducted to examine how the group averages differed according to the PA levels. The results are shown in Table 4.

**Table 4:** ANOVA results of mental health levels of participants according to physical activity levels

Scales	PAL	*n*	Mean	SS	F	*P*	Diff	η^2^
Psychological Resilience	**1-**Inactive	140	2.74	0.75	13.782	**0.001**	3 > 2 − 1	0.061
	**2-**Minimally Active	198	3.02	0.77				
	**3-**HEPA Active	89	3.26	0.70				
Mental Wellbeing	**1-**Inactive	140	3.29	0.65	13.390	**0.001**	3 > 2 − 1	0.059
	**2-**Minimally Active	198	3.45	0.61				
	**3-**HEPA Active	89	3.73	0.62				
Psychological Vulnerability	**1-**Inactive	140	3.04	0.72	6.652	**0.001**	1 > 2–3	0.030
	**2-**Minimally Active	198	2.81	0.75				
	**3-**HEPA Active	89	2.69	0.74				

*P* < 0.05; PAL = Physical Activity Levels

When the results obtained for the dependent variables were considered separately, according to the ANOVA results calculated at the level of 0.016 using Bonferroni adjusted alpha level, significant differences were found between the psychological resilience (F_(2.424)_ = 13.782, *p* = 0.001), mental well-being (F_(2.424)_ = 13,390, *p* = 0.001), and psychological vulnerability (F_(2.424)_ = 6.652, *p* = 0.001) scores of individuals depending on their PA levels. Based on the pairwise test results in all groups where significant differences were found, it was seen that the psychological resilience and mental well-being levels of the participants who were HEPA active were significantly higher than those of the participants in the minimally active and inactive groups. The findings also showed that the psychological vulnerability levels of the participants who were HEPA active were significantly lower than the ones in the minimally active and inactive groups.

### Findings related to hypothesis 3–5

Multiple regression analyses were performed to determine whether different types of PA were predictors of psychological resilience and mental well-being (Table 5).

**Table 5:** Multiple regression analysis fitting steps for the prediction of psychological resilience and mental wellbeing

	Unstandardized Coefficients		Standardized Coefficients			R^2^
	B	Std. Error	Beta	t	*p*	
		Psychological Resilience				
Walking	3.499	0.000	0.095	1.954	0.051	0.023
Moderate PA	5.411	0.000	0.074	1.499	0.135	
Vigorous PA	2.003	0.000	0.067	1.360	0.175	
		Mental Wellbeing				
Walking	5.509	0.000	0.177	3.703	0.000	0.045
Moderate PA	6.291	0.000	0.102	2.097	0.037	
Vigorous PA	1.447	0.000	0.006	0.118	0.906	

*p < 0.05

Multiple regression analysis revealed that physical activity levels significantly predicted psychological resilience and mental wellbeing. The psychological resilience model explained 2.3% of the variance (R² = 0.023), with walking showing an almost significant positive effect (B = 3.499, Beta = 0.095, *p* = 0.051), this suggests they may indicating a potential association between walking and resilience. Neither moderate physical activity (B = 5.411, Beta = 0.074, *p* = 0.135) nor vigorous physical activity (B = 2.003, Beta = 0.067, *p* = 0.175) showed statistically significant effects on resilince. In contrast, the mental wellbeing model explained 4.5% of the variance (R² = 0.045), with walking (B = 5.509, Beta = 0.177, *p* < 0.001) and moderate physical activity (B = 6.291, Beta = 0.102, *p* = 0.037) both showing significant positive associations, suggesting that increased walking and moderate physical activity are linked to better mental wellbeing. However, intense physical activity had no significant effect on mental wellbeing (B = 1.447, Beta = 0.006, *p* = 0.906).

Stepwise regression analyses were performed to reveal the effect levels of the types of physical activity found to be significant on psychological resilience and mental well-being (Table 6).

**Table 6:** Multiple regression (stepwise) analysis results for the prediction of psychological resilience and mental well-being

Independent variables	t	*R* ^2^	Adjusted *R*^2^	*R*^2^ change	Standardized b coefficient
	**Psychological Resilience**				
Model 1		0.012	0.009	0.012	
Walking	2.232*				0.108*
	**Mental Wellbeing**				
Model 1		0.034	0.032	0.034	
Walking	3.896*				0.186*
Model 2		0.045	0.041	0.011	
Walking	3.736*				0.178*
Moderate PA	2.164*				0.103*

*p < 0.05

In the first step of the stepwise regression analysis, the beta coefficient of the walking variable in predicting psychological resilience was measured as 0.108 and the “t test” result was found to be statistically significant (t = 2.232, *p* < 0.05). It was observed that 1.2% of the variance in the psychological resilience variable was estimated by participating in walking (R^2^ = 0.012; *p* < 0.05).

In the second step of the stepwise regression analysis, the beta coefficient of the walking variable in predicting mental well-being was measured as 0.186 and the “t test” result was found to be statistically significant (t = 3.896, *p* < 0.05). 3.2% of the variance in the mental well-being variable was estimated by participation in walking (Adjusted R^2^ = 0.032). Moderate PA was included in the model at the next step in the analysis process. When moderate PA performance was added, the result increased to 4.1% of the variance (Adjusted R^2^ = 0.041). The beta coefficient of the walking variable was found to be 0.178 and the beta coefficient of the moderate PA variable was measured as 0.103. The results of the “t test” were at a statistically significant level (t_walking_=3.736, *p* < 0.05/ t_moderate PA_=2.164, *p* < 0.05).

## Discussion

University life is known as a period during which students may face different problems such as moving away from their family, distress in adapting to new environments, being obliged to change some habits in terms of both comfort and health, academic pressures and emotional distress. In such situations, it is important for students to manage negative emotions such as vulnerability and to cultivate a peaceful lifestyle to support their mental health. In this study, the concept of mental health as a combination of both traditional psychology approach (psychological vulnerability) and positive psychology approach (mental well-being and resilience) was focused on and mental health was considered as the dependent variable. Physical activity, which is thought to contribute to the development of mental health, was analysed as the independent variable of the study. Physical activity not only reduces the risk of depression and anxiety problems, but also makes it easier for us to cope with what we are already experiencing [57]. This study examined how the levels and types of physical activity among university students influenced their mental health.

The results showed that 32.7% of the participants were inactive, 46.3% were minimally active and 20.8% were HEPA active. According to the current findings, 79% of the participants are not active enough in terms of physical activity participation. The results also revealed that female participants were more likely to be inactive and less likely to engage in intense activities compared to male participants. Based on these results, the proportional distribution of PA status according to grade level is similar. Descriptive findings suggested that students had relatively high levels of psychological vulnerability. Mental health components were also found to be interrelated. In other words, improvements in students’ mental well-being and psychological resilience levels may enable students to experience lower levels of vulnerability. Another important result obtained from the study is that students who are physically active enough have higher levels of mental well-being and psychological resilience and lower levels of psychological vulnerability. Finally, it was found that walking served as the strongest predictor of students’ psychological resilience and mental well-being. Furthermore, the level of mental well-being significantly increased when students engaged in moderate physical activity along with walking.

The results obtained from the survey have been discussed in line with the objectives of the research and within the scope of relevant literature.

Regarding the first objective, the findings have shown that gender and PA types are related. That is, it can be said that female students are more inactive than male students and male students engage in more intense physical activity than female students. The result that male participants were more likely to participate in intense activities is consistent with the study results of Granero et al. [32]. On the other hand, it was observed that there was no relationship between students’ grade level and PA types which means that students who differed in grade level participated in PA at relatively similar rates. However, consistent with this finding, Demirer and Erol, reported a significant relationship between gender and physical activity levels in their study [58]. In the current study, it has been concluded that psychological vulnerability is negatively and moderately related to mental well-being and psychological resilience while mental well-being and psychological resilience are positively and moderately related. These results support the **H**_**1**_ hypothesis. The suggestion that people with high levels of psychological vulnerability have lower psychological resilience and lower psychological coping skills is supported by current results [27]. In addition, it can be argued that students who feel relaxed and can make sound judgements have higher levels of achievements in terms of overcoming stressful events. As a matter of fact, in their meta-analysis study, Hu et al. stated that mental health is related to psychological resilience [28].

According to the findings related to the second objective of the current study, the students who were physically active enough had higher levels of mental well-being and psychological resilience compared to inactive and minimally active students. In other words, students who were physically active were more likely to be in a better condition in terms of some characteristics such as being optimistic about the future, feeling beneficial, and being able to recover quickly in distressed conditions compared to students with low PA levels. The results support the **H**_**2**_ and **H**_**4**_ hypotheses. These results are consistent with the view that physical activity frequency is significantly positively correlated with mental well-being, physical activity might reduce anxiety levels, and moreover, regular participation in physical activity may improve the overall health and mental well-being [31, 59, 60]. With respect to the findings, physically inactive students were characterised as experiencing higher levels of vulnerability compared to minimally and HEPA active students. In another word, students with low levels of physical activity experienced significantly higher levels of emotions such as feeling worthless, needing approval, and experiencing disappointment than those with moderate and high levels of physical activity. The results support the **H**_**6**_ hypothesis. The argument that depressive symptoms are and will continue to be effective on university students [23] emphasises the importance of participation in PA once again.

Findings related to the third and final objective showed that walking had an effect on students’ psychological resilience and mental well-being. In short, walking is the best predictor of students’ mental well-being and resilience. At the same time, when moderate intensity activities are added to the walking, students are likely to feel more comfortable and cope with problems more easily. These results supported hypothesis **H**_**3**_, while hypothesis **H**_**5**_ was not confirmed. Even low levels of physical activities have positive effects on students’ well-being. It is stated that only 60 min of physical activity per week reduces depression by 12% [61]. Therefore, the findings obtained from the study were consistent with the benefits of engaging in moderate-intensity physical activity, particularly walking. While Herbert stated that studies involving low to moderate intensity aerobic exercises for a few weeks could improve the mental health of university students [33], Dunston et al. suggested that intense physical activity was positively associated with psychological resilience scores of university students [36]. However, these findings show that there is no consensus in the literature about which type of PA is more beneficial. In fact, from high- intensity activities (such as aerobic and strength training) to walking, all types of physical activity are beneficial [25].

### Implications and limitations

The study provided a holistic approach by examining mental health components such as psychological vulnerability, mental well-being and psychological resilience together. In addition, by determining the effects of walking and moderate physical activities on mental health, original and practical results were obtained in these areas. The fact that the participants were selected from a wide demographic group and the importance of physical activity was emphasised based on current data makes the study more valuable. This research has some limitations. First of all, it is not possible to establish a causal relationship between variables in cross-sectional studies. Therefore, longitudinal studies can reveal the relationships between variables more clearly. In addition, in the present study, physical activity, the independent variable of the study, was measured by indirect methods (a questionnaire). This may have caused some subjective biases. The fact that the studies were not carried out on the same date can be considered as a limitation of this research. Moreover, the relationships between the variables in the study may be affected by the gender and age of the students. In particular, the unequal distribution of women and men is a limitation of the study. Finally, although statistically significant, the low variance explained in the regression analysis is considered a limitation in terms of the generalizability of the data.

### Recommendations

Students are advised to participate in activities that require moderate physical exertion, especially regular walking, cycling at a normal speed, dancing, playing tennis, folk dances or bowling. In this way, university students can improve and nourish their mental health. Considering the relatively high averages in the vulnerability levels, it is thought that improvement (guidance and psychological support) activities can be beneficial for students. In order to increase the generalizability of the research findings, studies can be conducted on more students studying at public and private universities. Considering the limitations of convenience sampling method, it is recommended to prefer probability sampling methods in future research. This research was arranged in a cross-sectional structure. In future studies, longitudinal structures in different sample groups can be practised to examine the role of physical activity on mental health.

## Conclusions

This study sheds light on the relationship between university students’ physical activity levels and their mental health. Findings suggest that participation in physical activity not only reduces depression and anxiety levels, but also significantly increases individuals’ psychological resilience and mental well-being. In particular, walking was found to have one of the strongest effects on students’ mental resilience and well-being, and this effect was further enhanced when combined with moderate physical activities. While the study reveals that female students participate in less physical activity and have higher levels of psychological vulnerability compared to male students, it draws attention to the significant effects of this situation on mental health. However, it has been revealed that participation in physical activity, even at a low level, contributes to students’ optimistic view of the future, improving their ability to cope with stress and reducing their psychological vulnerability. In order to improve the quality of life of university students and to support their mental health, it is a necessity to promote participation in physical activity.

## Data Availability

The data sets generated during and/or analyzed during the current study are available from the corresponding author on reasonable request.

## Abbreviations

WHO: World Health Organization
PA: Physical Activity
HEPA: Health Enhancing Physical Activity
MET: Metabolic Equivalent of Task
IPAQ-SF: International Physical Activity Questionnaire-Short Form
WEMWB-SF: Warwick-Edinburgh Mental Wellbeing Scal- Short Form
PR: Psychological Resilience
MWB: Mental Wellbeing
PV: Psychological Vulnerability
PAL: Physical Activity Levels
